# A simplified approach to estimating the distribution of occasionally-consumed dietary components, applied to alcohol intake

**DOI:** 10.1186/s12874-016-0178-3

**Published:** 2016-07-01

**Authors:** Julia Chernova, Ivonne Solis-Trapala

**Affiliations:** 1 MRC Human Nutrition Research, Elsie Widdowson Laboratory, Cambridge, UK; 2 Institute for Applied Clinical Sciences, Keele University, Staffordshire, UK

**Keywords:** Excess-zeros, Semi-continuous, Two-part model, Random effects, Repeated measurements, Alcohol, Quantiles

## Abstract

**Background:**

Within-person variation in dietary records can lead to biased estimates of the distribution of food intake. Quantile estimation is especially relevant in the case of skewed distributions and in the estimation of under- or over-consumption. The analysis of the intake distributions of occasionally-consumed foods presents further challenges due to the high frequency of zero records. Two-part mixed-effects models account for excess-zeros, daily variation and correlation arising from repeated individual dietary records. In practice, the application of the two-part model with random effects involves Monte Carlo (MC) simulations. However, these can be time-consuming and the precision of MC estimates depends on the size of the simulated data which can hinder reproducibility of results.

**Methods:**

We propose a new approach based on numerical integration as an alternative to MC simulations to estimate the distribution of occasionally-consumed foods in sub-populations. The proposed approach and MC methods are compared by analysing the alcohol intake distribution in a sub-population of individuals at risk of developing metabolic syndrome.

**Results:**

The rate of convergence of the results of MC simulations to the results of our proposed method is model-specific, depends on the number of draws from the target distribution, and is relatively slower at the tails of the distribution. Our data analyses also show that model misspecification can lead to incorrect model parameter estimates. For example, under the wrong model assumption of zero correlation between the components, one of the predictors turned out as non-significant at 5 % significance level (*p*-value 0.062) but it was estimated as significant in the correctly specified model (*p*-value 0.016).

**Conclusions:**

The proposed approach for the analysis of the intake distributions of occasionally-consumed foods provides a quicker and more precise alternative to MC simulation methods, particularly in the estimation of under- or over-consumption. The method is readily available to non-technical users in contrast to MC methods whereby the simulation error may be substantial and difficult to evaluate.

**Electronic supplementary material:**

The online version of this article (doi:10.1186/s12874-016-0178-3) contains supplementary material, which is available to authorized users.

## Background

Monitoring usual or long-term dietary intake is of interest to health researchers and public health policy makers to assess nutrient adequacy of a group or population. Recent public-health programmes include monitoring of alcohol consumption by personal, social and demographic characteristics in the research programme “Reducing alcohol-related health harms in an English context” led by the School for Public Health Research of the UK National Institute for Health Research [36]; and folate consumption in child-bearing age women and birth defects [34].

The statistical analysis of dietary data presents several challenges due to limitations in dietary assessment tools and the presence of within-person variation in consumption. The most commonly used dietary assessment tools are food frequency questionnaires (FFQ), food diaries (FD) and 24 hour food recalls (24HR). Of these, methods comparison and biomarker validation studies suggest that multiple days FD and multiple 24HR are more reliable [4, 5, 8, 9, 18].

These tools were developed to capture long-term habitual diet but due to reduced observation periods, they are subject to observational error, defined as the difference between the measured diet and its true value [3, 25]. Moreover, the records of intake of occasionally-consumed dietary components (e.g. fish, alcohol, nuts) usually contain high frequencies of zeros, adding further complexity to the analysis of the distribution of these components. The mean and a measure of spread describe symmetrical distributions well, but not those with skewed shapes. The majority of occasionally-consumed food intake distributions have skewed shapes so the information contained in the mean and a measure of spread will not suffice to estimate, say, under- or over- consumption, which is often of major interest to public health policy makers. Therefore, in the evaluation of dietary intake, the tails of the population intake distribution are often as important as the mean or the median. Thus, quantile estimation provides a useful tool for monitoring diet and complements regression analysis of the mean.

This paper proposes a numerical approach to estimate the quantiles of the distribution of occasionally-consumed foods in specified sub-populations. Our method accounts for within-person variation, correlation arising from multiple measurements taken from the same person and the high frequency of zero observations of recorded food intake.

### Within-person variation

Within-person variation arises from individual daily variation in food consumption and observational error. The mean of observed individual dietary records is often used as a measure of true individual intake; however, the mean contains information of both, the true long-term habitual intake and within-person variation. Although increasing the number of days in dietary records reduces observational error [21], in practice, most FDs and 24HRs contain only 2 to 4 days of dietary intake records, which leads to a significant daily variation in individual means [3, 20, 26]. Therefore, using individual means to describe food intake in population groups artificially inflates the group variance estimate, which, in turn, results in biased estimates of upper and lower quantiles of food intake distribution and in biased estimates of compliance with respect to recommended intake guidelines [13, 33]. To illustrate this, consider the estimation of the 90th quantile of a normal distribution. If the mean is 0 and the standard deviation 1, the 90th quantile is 1.28. But, the same 90th quantile, for a distribution with the same mean, but 1.5 times larger standard deviation becomes 1.92.

Dodd et al. [11] provided a review of statistical methods which account for within-person variation when estimating the distribution of usual dietary intake within a population group using individual means. More recently, [33] suggested utilising a mixed-effects modelling approach without reducing the data to individual averages. This method suggests that if a person *i* has true intake *T*_*i*_ ($T^{*}_{i}$ on a transformed scale) then the individual daily food record *R*_*ij*_ ($R^{*}_{ij}$ on a transformed scale), of a person *i* on day *j*, can be described as *R*_*ij*_=*T*_*i*_+*ε*_*ij*_ ($R^{*}_{ij}=T^{*}_{i}+\epsilon _{ij})$, where *ε*_*ij*_ represents random daily variation and is assumed to have mean 0 and variance $\sigma ^{2}_{\epsilon }$. This assumption can be described as the unbiasedness of the recorded individual intake either on the original or a transformed scale. Then the total group variance of food intake distribution is decomposed into a within-person ($\sigma ^{2}_{\epsilon }$, daily) and between-person (true) parts. Using the estimated between-person variance and mean and assuming approximately Gaussian distribution of food intake distribution (on the original or transformed scale), we can reconstruct the true food intake distribution within a specified group leaving out the estimated within-person variance. Several applications of this method can be found in the literature [13, 33].

### Excess zeros

Occasionally consumed foods are further characterised by high frequency of zero intake records, which presents further challenges in analysis. Firstly, the methods of dealing with within-person variance described above are not directly applicable to zero-inflated data as they assume that food intake can be transformed to be approximately Gaussian using a monotone function. This distributional assumption is clearly violated for occasionally consumed foods. Secondly, the number of daily records needed to reliably estimate within-person and between-person variation, if consumption occurs only infrequently, exceeds the number of daily records typically available from food diaries or food recalls.

A preferred method for modelling occasionally-consumed food intake for a given individual, adopted in this paper, looks at the data as generated by a two-step process: the first step (the *probability* step) generates the event of consumption (yes/no) on a given day and the second step (the *amount* step) generates the amount of food consumed on a consumption day. The probability part can be modelled by a mixed-effects logistic regression and the amount component by a mixed-effects linear regression model.

Importantly, as discussed by [23] and [30], consumption behaviours are complex and the outcomes of the first and the second steps are not, generally, independent. In particular, it is plausible that the more often someone consumes, the larger the amount consumed on any given consumption day: examples include fruits and vegetables, whole grains and alcohol [2, 32]. Consequently, the *probability* and the *amount* parts are likely to be correlated.

The correlation can arise, *inter alia*, from personal preferences affecting the probability of consumption and the amount consumed simultaneously. When some of these personal preferences are unobserved, because they may be impractical, impossible, or very expensive to measure, the model needs to account for this unobserved heterogeneity. This can be done through inclusion of one random effect into each component of the model and allowing the two random effects to be correlated. Ignoring this correlation in the estimation of food intake distribution when, in fact, the correlation is positive, can lead to over-estimation of the amount consumed by people with low probability of consumption and under-estimation of the amount consumed by people with high probability of consumption. The magnitude of the bias can be especially pronounced when the between-person variation is quite large and there is not enough information to explain it and when the correlation between unobserved preferences is substantial [1, 16, 27].

Monitoring dietary intake at a group level requires the estimation of distribution characteristics, such as quantiles. Obtaining these from the two part mixed-effects model is not straightforward due to the presence of the random effects in the model. The current practice, suggested by [32], is to: i) estimate individual linear predictors from fitting the two-part model, ii) simulate 100 random effects, per individual, from a bivariate normal distribution, with mean zero and variance parameters estimated from the fitted model, iii) add the simulated random effects to the estimated linear predictors, and iv) obtain empirical quantile estimates from the simulated datasets. This method forms part of the NCI method [30, 32] for the estimation of usual dietary intake, recommended by the US National Institute of Health. However, the precision of MC estimates is affected by random sampling variation, and the size of the simulated data that is needed to achieve the required precision is population- and model-specific, which can hinder reproducibility of results. The simulations can also be time consuming with increasing number of sub-populations for which intake distribution is of interest.

We suggest an approach which is based on the two-part model [23, 30] and circumvents the need of simulation by use of numerical integration to estimate the distribution of occasionally-consumed food in specified sub-populations. The method is a quicker, easier to implement and more accurate alternative to the simulation-based method. Additionally, we illustrate the impact of ignoring the correlation between the *probability* and *amount* parts of the two-part model in the model specification, and compare the performance of our approach with that based on Monte Carlo (MC) simulations.

## Methods

In this section we describe the two-part mixed-effects model [23, 30] for modelling individual intakes of occasionally-consumed foods. We then show how this model is utilised to estimate the distribution of habitual dietary intake in sub-populations, whereby the individual *true* expected intake is estimated as the product of the probability of consumption times the expected amount consumed. Finally, we describe the proposed method for the quantile estimation of habitual dietary intake.

### Two-part mixed-effects model

We briefly describe the two-part mixed-effects model for repeated positive continuous responses with excess zeroes (cf. [23, 27, 30] for full details). As discussed above, for each person, *i*,*i*=1,…,*m* on day *j*,*j*=1,…*n*_*i*_, the data consist of two parts: the occurrence of food consumption (yes/no), which can be recorded as an indicator variable *I*_*ij*_ such that:

$$ I_{ij} = \left\{ \begin{array}{ll} 1, & \text{if the food is consumed by person \textit{i} on day \textit{j}} \\ 0, & \text{otherwise} \end{array}\right. $$

and the amount of food consumed if consumption took place, which we record as *A*_*ij*_,*A*_*ij*_>0 if *I*_*ij*_=1.

Natural heterogeneity arise among subjects due to personal preferences for consumption. We denote unobservable person-specific information related to propensity to consume certain foods as *v*_*i*_ and unobservable person-specific information related to amount consumed on consumption day as *u*_*i*_. Then, conditionally on *v*_*i*_ and *u*_*i*_, responses *I*_*ij*_ and *A*_*ij*_ are independent. The indicator variable *I*_*ij*_ is assumed to follow a Bernoulli distribution with probability *p*_*ij*_, and to allow for skewness, we assume *A*_*ij*_,*A*_*i*,*j*_>0 to be log-normally distributed. In this paper, we suggest the following model specification: the first part response *I*_*ij*_ follows the logistic regression model:

$$ \operatorname{logit} \{\operatorname{Pr}(I_{ij}=1|v_{i}) \}= x_{ij}^{'}\gamma+v_{i} $$

where $x_{ij}^{'}$ is the vector of relevant covariates, relating individual characteristics to propensity for food intake, and *γ* is the vector of corresponding regression coefficients. And, considering, log(*A*_*ij*_)=*Y*_*ij*_ is approximately normal, we can write:

$$Y_{ij}=x_{ij}^{'}\beta+u_{i}+\epsilon_{ij} $$

where $ \operatorname {E}(Y_{ij}|u_{i})=x_{ij}^{'}\beta + u_{i}$ and $\operatorname {Var}(Y_{ij}|u_{i}) = \sigma _{\epsilon }^{2}$ (within-person daily variation); $x_{ij}^{'}$ is the vector of relevant covariates relating individual characteristics to the amount of food consumed, *β* is the vector of corresponding regression coefficients. The potential correlation between the *probability* and *amount* parts is linked through person-specific effects *u*_*i*_ and *v*_*i*_, which are assumed to have a common bivariate normal distribution with means 0 and variance-covariance matrix:

$$\Sigma = \left(\begin{array}{cc} {\sigma_{u}^{2}} & \rho\sigma_{u}\sigma_{v} \\ \rho\sigma_{u}\sigma_{v} & {\sigma_{v}^{2}}\\ \end{array} \right) \; $$

where *ρ* denotes the correlation between *u*_*i*_ and *v*_*i*_, ${\sigma _{u}^{2}}$ and ${\sigma _{v}^{2}}$ are the variances of *u*_*i*_ and *v*_*i*_ respectively. These are called random effects and are assumed to be independent of *ε*_*ij*_. The unknown model parameters *θ*=(*γ*,*β*,*σ*_*u*_,*σ*_*v*_,*σ*_*ε*_,*ρ*) can be estimated through maximising the full marginal likelihood function, where we utilise the conditional independence of responses *I*_*ij*_ and *Y*_*ij*_ and their distributional assumptions. Because the random effects *u*_*i*_ and *v*_*i*_ are unobserved, they need to be integrated out, so that the full marginal likelihood function is:

1 $${} \begin{aligned} L(\theta) \propto \prod_{i=1}^{m} \int_{-\infty}^{+\infty} \int_{-\infty}^{+\infty} \prod_{j=1}^{n_{i}} f_{I}(I_{ij}\mid v_{i}, \theta)f_{Y}(Y_{ij}\mid u_{i}, \theta)\\[-5pt] f_{UV}(u_{i}, v_{i}\mid \theta)du_{i}dv_{i} \end{aligned}  $$

where *f*_*I*_, *f*_*Y*_ and *f*_*UV*_ denote the density functions of the binomial, normal and bivariate normal distributions, respectively. The likelihood function does not have a closed form and needs to be evaluated numerically. We note that if it is assumed that the random effects are independent, i.e. *ρ*=0, estimation is considerably simplified as the two parts can be fitted separately using standard statistical software for generalised mixed effects models. However, if this assumption does not hold, i.e. *ρ*≠0, then the estimation of the two-part model requires more specialised programming, for example, the SAS PROC NLMIXED procedure (SAS Institute, Cary, NC, Version 9.1, Littell *et al* 2006, SAS for mixed model) can be used in this case.

### Distribution of habitual dietary intake

The expected individual habitual daily intake *T*_*ij*_ for a person *i* on a day *j* is calculated as the product of the individual daily probability of consuming the food, *p*_*ij*_, and the expected individual consumed amount on a consumption day:

*T*_*ij*_=*P*(*I*_*ij*_=1|*v*_*i*_)·*E*(*A*_*ij*_|*A*_*ij*_>0,*u*_*i*_). Under the two-part model *T*_*ij*_ depends on the regression parameters *β* and *γ*, as well as the unobserved person-specific effects *u*_*i*_ and *v*_*i*_, which may be correlated. Maximum likelihood estimates: $\tilde {\beta }, \tilde {\gamma }, \tilde {\Sigma }, \tilde {\sigma _{\epsilon }}$ can be obtained by fitting the two-part model, but the person-specific variation has to be accounted for when estimating a group distribution of dietary intake. One way to account for this variation is to perform MC simulations.

This method and its application in the present context has been described elsewhere [12,30,32]. Briefly, first, point estimates of the model parameters are obtained from fitting the two-part model. Secondly, for each combination of covariates of interest, fixed effect predictions are obtained using the estimated regression coefficients. Thirdly, N pairs (*u*_*i*_,*v*_*i*_) are generated from a bivariate normal distribution with the parameters of the distribution estimated earlier at the first step. Tooze et al. [32] recommends to simulate 100 observations per original sample observation with the same covariate values but varying person-specific effects. Thus, for each combination of covariates we have a dataset containing N (e.g. 100 times the original sample size) simulated observations whose distribution characterises the distribution of occasionally-consumed dietary intake in a sub-population with the same covariate pattern as that of the observed sample. This dataset is then used to obtain empirical quantile estimates. If the intake is assumed to be unbiased on the original scale then back-transformation needs to be used [22].

This paper suggests the use of optimisation and numerical integration methods to estimate the quantiles of occasionally-consumed food intake distributions as an alternative to MC simulations. To compare the proposed approach with MC simulations, we undertook a simulation study following the NCI method described above, up to the point where we needed to decide on the size of simulated data. One of the research questions we set to answer was to investigate the MC convergence in the context of the application of the two-part model, so it was decided to simulate data sets of varying size including 1000, 5000, 10000 and 50000 observations per fixed covariate values. The covariates we adjusted for in the model were gender and age, so for men and women, and for each of the following age values (years): 40, 45, 50, 55, 60, 65 we simulated 4 data sets of different sizes. In the Results section we compare how our MC simulated results compare with the results obtained from the proposed approach. The following section describes the proposed numerical method.

#### Quantiles of habitual dietary intake

Quite often, the distribution of the amount of food consumed on a consumption day appears to be skewed and a logarithmic transformation can be an appropriate choice to obtain a symmetric distribution [35]. If we assume that the individual transformed intake *Y*_*ij*_|*u*_*i*_ follows a normal distribution with expectation $x_{ij}^{'}\beta +u_{i}$ and variance *σ*_*ε*_ then *A*_*ij*_|*u*_*i*_ follows log-normal distribution with expected value $\exp \left (x_{ij}^{'}\beta +u_{i}+0.5\sigma _{\epsilon }^{2}\right)$ so we can write down the individual expected daily marginal amount consumed as

$$\tilde{T_{ij}}=\exp\left(x_{ij}^{'}\tilde{\beta}+u_{i}+0.5\sigma_{\epsilon}^{2}\right)\frac{\exp\left(x_{ij}^{'}\tilde{\gamma}+v_{i}\right)}{1+\exp\left(x_{ij}^{'}\tilde{\gamma}+v_{i}\right)} $$

Dietary intake, alcohol consumption for example, is likely to vary between a week day and a weekend. To account for this, the expected weekly consumption $\tilde {T}_{i}$ is estimated as the weighted average of habitual daily consumption comprising 4 working-week days and 3 weekend days:

$${} \begin{aligned} \tilde{T_{i}}&=4\exp\left(x_{i0}^{'}\tilde{\beta}+u_{i} + 0.5\sigma_{\epsilon}^{2}\right)\frac{\exp\left(x_{i0}^{'}\tilde{\gamma}+v_{i}\right)}{1+\exp\left(x_{i0}^{'}\tilde{\gamma}+v_{i}\right)}\\ &\quad+3\exp\left(x_{i1}^{'}\tilde{\beta}+u_{i}+ 0.5\sigma_{\epsilon}^{2}\right)\frac{\exp\left(x_{i1}^{'}\tilde{\gamma}+v_{i}\right)}{1+\exp\left(x_{i1}^{'}\tilde{\gamma}+v_{i}\right)} \end{aligned} $$

where $\tilde {\beta }$ and $\tilde {\gamma }$ are point estimates from the two-part model, $x_{i0}^{'}$ are covariates corresponding to a working-week day and $x_{i1}^{'}$ are covariates corresponding to a weekend. $\tilde {T}_{i}$ depends on the two random variables *u*_*i*_ and *v*_*i*_. By definition of cumulative distribution function, for a given probability *p* and the corresponding quantile *c*_*p*_, we can write:

2 $$ P(\tilde{T_{i}} \leq c_{p})=p   $$

which, when substituting $\tilde {T_{i}}$, is equivalent to

$$\begin{aligned} &P\left(4\exp(x_{i0}^{'}\tilde{\beta}+u_{i}+ 0.5\sigma_{\epsilon}^{2})\frac{\exp(x_{i0}^{'}\tilde{\gamma}+v_{i})}{1+\exp(x_{i0}^{'}\tilde{\gamma}+v_{i})}\right. \\ &\left.+ 3\exp(x_{i1}^{'}\tilde{\beta}+u_{i}+ 0.5\sigma_{\epsilon}^{2})\frac{\exp(x_{i1}^{'}\tilde{\gamma}+v_{i})}{1+\exp(x_{i1}^{'}\tilde{\gamma}+v_{i})} \leq c_{p}\right) = p \end{aligned} $$

After re-arranging the terms and taking natural logarithm, the above expression is equivalent to

$$\begin{aligned} &P\left(u_{i} \leq \ln(c_{p})- \ln \left\{ 4\exp(x_{i0}^{'}\tilde{\beta}+ 0.5\sigma_{\epsilon}^{2})\frac{\exp(x_{i0}^{'}\tilde{\gamma}+v_{i})}{1+\exp(x_{i0}^{'}\tilde{\gamma}+v_{i})}\right.\right.\\ &\left.\left. \quad\qquad+ 3\exp(x_{i1}^{'}\tilde{\beta}+ 0.5\sigma_{\epsilon}^{2})\frac{\exp(x_{i1}^{'}\tilde{\gamma}+v_{i})}{1+\exp(x_{i1}^{'}\tilde{\gamma}+v_{i})} \right\} \right) = p \end{aligned} $$

Let *h*(*c*,*v*_*i*_) denote the function:

$$\begin{aligned} h(c,v_{i}) \equiv \ln(c)&-\ln \left(4\exp(x_{i0}^{'}\tilde{\beta}+ 0.5\sigma_{\epsilon}^{2})\frac{\exp(x_{i0}^{'}\tilde{\gamma}+v_{i})}{1+\exp(x_{i0}^{'}\tilde{\gamma}+v_{i})}\right.\\ &\left.+3\exp(x_{i1}^{'}\tilde{\beta}+ 0.5\sigma_{\epsilon}^{2})\frac{\exp(x_{i1}^{'}\tilde{\gamma}+v_{i})}{1+\exp(x_{i1}^{'}\tilde{\gamma}+v_{i})} \right) \end{aligned} $$

Then under the distributional assumptions for *v*_*i*_ and *u*_*i*_ as bivariate normal (0,*Σ*) we can re-write (2) as

3 $$ \int_{-\infty}^{+\infty} \int_{-\infty}^{h(c,v_{i})} f_{BN}(u_{i}, v_{i})du_{i}dv_{i} = p.   $$

The solution of (3) with respect to *c*, is the quantile *c*_*p*_, corresponding to a given probability *p*. Additional file 1: Appendix 1 shows why this solution exists and is unique under the given model assumptions. To find *c*_*p*_ the integral in (3) can be approximated numerically, e.g. by quadrature methods, and the solution to the equation found through optimisation. The implementation of this in R is available from the authors upon request.

### Data

To illustrate the method, we analysed alcohol intake from the screening phase of the RISCK (Reading, Imperial, Surrey, Cambridge, and Kings) study [14], which is a randomised controlled trial (RCT), investigating the effect of the types of fats and carbohydrates in diet on glucose and insulin metabolism. Participants were recruited from the general population and baseline measures were collected from August 2004 to April 2006. The participants were eligible if their weight was stable 3 months prior to enrolment, i.e. their energy intake and energy expenditure were in balance [24], and if they were at risk of developing metabolic syndrome with special emphasis on enrolling participants with impaired glucose tolerance. Initially, 7-day food diaries were collected from 531 participants. These yielded 2214 days of dietary records in total, with the majority (81 %) providing 4 days of the foods records. However, to reduce potential bias in data analysis [7,19,31], this analysis excludes data from 209 (39 %) participants due to extreme under-reporting, leaving for analysis data on 322 (61 %) participants. The status of *under-reporters* was defined by the Goldberg cut-off [6] (see Additional file 2: Appendix 2 for further details). Ethical approval for the RISCK study was obtained from the National Research Ethics Service, and written informed consent was given by participants.

## Results

### Descriptive analysis

The sample available for analysis consists of 186 (58 %) women, with the following characteristics summarised as mean (standard deviation) or frequency (%): age 52 years (10), body mass index (BMI) 27.5 (4.2), smoking status (yes) 31 (4 %), degree of under-reporting 0.96 (0.15); and of 136 (42 %) men: age 53 years (11), BMI 27.6 (3.4), smoking status (yes) 36 (6.3 %) and degree of under-reporting 0.93 (0.13).

To describe the probability of consuming alcohol in the period of observation, the ratio of the number of reported alcohol consumption days over the total number of diary records available for each participant was calculated. Table 1 shows that men and women have significantly different consumption patterns (overall *p*-value from chi-squared test is 0.004): more women than men (70 (37.6 %) versus 32 (23.5 %)) reported no alcohol consumption, whereas, there are fewer women than men (26 (14.0 %) versus 32 (23.5 %)) whose estimated probability of consuming is greater than 0.75 on a given day.

**Table 1:** Percentage of days of recorded alcohol intake out of total recorded days available

Percentage of days with recorded alcohol consumption	Men, N (%)	Women, N (%)
0 records	32 (23.5)	70 (37.6)
>0 and ≤0.25	20 (14.7)	42 (22.6)
>0.25 and ≤0.5	27 (19.9)	26 (14.0)
>0.5 and ≤0.75	25 (18.4)	22 (11.8)
>0.75	32 (23.5)	26 (14.0)

Percentage of days of recorded alcohol intake was estimated as a ratio of the number of reported alcohol consumption days over the total number of diary record days available

**Fig. 1:**
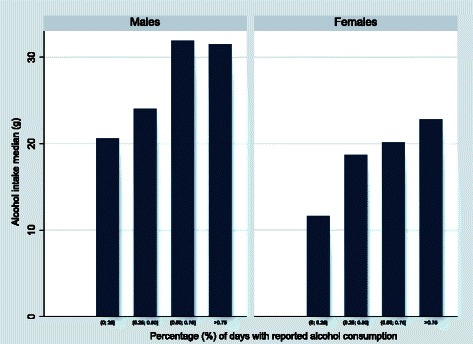
Percentage of reported alcohol days and median of alcohol intake. The bar graphs show how the group median amount of alcohol consumed (g) on consumption day (estimated from individual averages) increases with increasing percentage of reported alcohol days. Percentage of reported alcohol days is calculated as the ratio of the number of reported alcohol consumption days over the total number of diary record days available and split into 5 categories

Despite different frequency patterns of alcohol consumption, both, men and women, tend to consume more alcohol on a given consumption day if their frequency of consumption is higher compared to those who consume less frequently (Fig. 1).

### Modelling alcohol intake

The statistical analyses were stratified by sex. After preliminary screenings of the sampling distributions of alcohol intake on consumption days, a logarithmic transformation was adopted to obtain a more symmetric distribution of the data. Figure 1 suggests that there might be a positive correlation between the probability of consuming alcohol and the amount of alcohol consumed on consumption day. We fitted the two-part mixed-effects model assuming that the correlation between the two parts is positive (Model A) and assuming that the correlation is zero (Model B). We compare the analysis results from Models A and B to assess the impact of model misspecification on both the estimation of parameters related to individual alcohol intake and the distribution of alcohol intake in specified sub-groups. We note that the regression parameters in the two parts of the models are person specific.

#### Correlation between probability and amount parts of the model

The estimated (adjusted for age and weekend) correlation between the model parts is 0.55 (*p*-value 0.004) in females and 0.30 (*p*-value 0.160) in males. This suggests that there exist some person-specific characteristics which simultaneously increase the probability to consume alcohol and the amount of alcohol consumed on consumption day.

#### Probability part

Estimates show no difference between models A and B in the estimation of the odds of daily alcohol consumption for both groups, men and women (Table 2).

**Table 2:** Effect of the covariates on daily probability of alcohol consumption and amount of alcohol consumed

Males Females				
Probability part	Odds ratio	*p*-value	Odds ratio	*p*-value
	95 %CI		95 %CI	
Model A				
Weekend	3.95	<0.001	3.56	<0.001
	(2.37, 6.60)		(2.27, 5.56)	
5 years increase in age	1.27	0.042	1.29	0.031
	(1.01, 1.54)		(1.03, 1.55)	
Model B				
Weekend	3.99	<0.001	3.53	<0.001
	(2.39, 6.66)		(2.25, 5.54)	
5 years increase in age	1.27	0.043	1.30	0.028
	(1.01, 1.53)		(1.03, 1.56)	
Amount part	Ratio of change	*p*-value	Ratio of change	*p*-value
	95 %CI		95 %CI	
Model A				
Weekend	1.48	<0.001	1.28	0.016
	(1.23, 1.79)		(1.04, 1.56)	
5 years increase in age	0.96	0.162	1.00	0.910
	(.90, 1.02)		(0.91, 1.08)	
Model B				
Weekend	1.45	0.001	1.23	0.062
	(1.19, 1.73)		(0.99, 1.48)	
5 years increase in age	0.95	0.105	0.98	0.695
	(0.89, 1.01)		(0.90, 1.06)	
Correlation between				
probability and	0.30	0.160	0.55	0.004
amount parts				

Model A adjusts for correlation between *probability* and *amount* parts

Model B assumes zero correlation between *probability* and *amount* parts

#### Amount part

The regression parameters are interpreted as percentage change in the amount of alcohol consumed on consumption day with a unit-change in the corresponding covariate, holding the other covariates fixed. For females, model B shows *weekend* as a non-significant predictor (at 5 % significance level): 1.23 (95 %CI (0.99, 1.48), *p*-value 0.062) times increase in the amount of alcohol consumed given consumption took place on weekend compared to a week day; whereas model A shows that, on weekend, women increase the amount of alcohol consumed (given it was consumed) by 1.28 times (95 %CI (1.04, 1.56), *p*-value 0.016). Thus, under the wrong assumption of zero correlation between the model parts, a statistically significant predictor turns into non-significant.

For males the discrepancy between the results obtained from model A and model B is not as pronounced: 1.45 times increase on *weekend* in amount consumed if consumption takes place (95 %CI (1.19, 1.73), *p*-value 0.001) for model B, and 1.48 times increase (95 %CI (1.23, 1.79), *p*-value <0.001) for model A.

These findings show that when the zero correlation assumption between *probability* and *amount* parts is strongly violated, model A provides better estimates of regression coefficients. However, the greatest discrepancies between the results from model A and model B tend to be observed not around the (geometric) mean but around the tails of the distribution of alcohol intake.

### Distribution of weekly alcohol consumption

Table 3 shows the magnitude of discrepancies between weekly alcohol intake distributions estimated under model A and B assumptions, separately for males and females and for various ages, for the following quantiles: 0.1, 0.25, 0.50, 0.75, 0.90 and 0.95. The difference between the models is most obvious at the tails of the distribution, where Model A, as expected from the theory, gives higher estimates than model B for higher quantiles. For example, our data show that, in men, model A estimates 0.90 quantile to be 321.8g versus 301.6g (model B) of weekly alcohol intake in 40-year-old participants. Since the detrimental effect of alcohol is believed to arise from excessive consumption, our results demonstrate that the application of the model with the correct assumptions provides a more accurate assessment of the potential public health burden.

**Table 3:** Alcohol intake quantiles estimates

Quantiles							
Age, y	Model	0.1	0.25	0.5	0.75	0.9	0.95
Men							
40	A	8.2	29.6	91.1	194	321.8	419.1
	B	11.1	36	97.7	190	301.6	386
	MC1	8.6	32	97.1	190.3	311.4	407.7
	MC5	8.2	29.5	91.3	191.5	312.4	410.9
	MC10	7.9	29.7	91	192	322.7	420.2
	MC50	8.3	30.1	90.6	194.1	321.8	416.2
45	A	9.8	34.2	97.9	198.1	321.3	414.9
	B	13.2	41.1	103.9	193.4	301.2	382.8
	MC1	7.8	32.6	86.1	190.4	306.4	391.5
	MC5	9.7	34.2	96	199.7	317.6	408.4
	MC10	9.4	33.9	97.3	196.1	317.3	416.4
	MC50	9.7	34.6	98	198.6	322.5	414
50	A	11.7	39	104	201	319.5	409.6
	B	15.5	46.3	109.2	195.6	299.6	378.5
	MC1	12.5	43.1	108	199.5	330.1	386.3
	MC5	13	41.3	104.9	203.1	325.1	416.6
	MC10	11.2	37.9	101.7	201.3	320.9	413.3
	MC50	11.4	38.5	103.9	201.7	317.4	407.8
55	A	13.9	43.9	109.2	202.7	316.6	403.2
	B	18.2	51.5	113.6	196.8	297.1	373.2
	MC1	13.5	44.5	108.5	202	301.7	396.1
	MC5	13.3	42.4	107	205.4	318.7	406.4
	MC10	13.5	42.1	105.2	198.4	308.5	390.1
	MC50	13.7	43	108.5	204	319.7	406.8
60	A	16.3	48.9	113.5	203.3	312.7	395.9
	B	21.1	56.8	117	197	293.7	367.1
	MC1	14.5	43.9	107.6	200.4	306.7	375.8
	MC5	15.3	48.7	113.7	203.9	319	408
	MC10	16.4	47.7	111.2	203.2	311.1	392.1
	MC50	15.9	48.9	114.5	204.8	314.7	397.6
65	A	19	53.7	116.9	203	307.9	387.9
	B	24.5	61.5	119.6	196.4	289.4	360.2
Quantiles							
Age, y	Model	0.1	0.25	0.5	0.75	0.9	0.95
	MC1	19.4	55	120.4	197.7	303.9	370.1
	MC5	18.5	52.6	115.9	201.8	304	392
	MC10	19.7	54.2	117.6	202.8	311.4	394.1
	MC50	19.2	54.3	118	204.3	308	387.1
Women							
40	A	0	4	20.1	71.7	166	251
	B	1.7	6.5	25.2	70.7	143.5	207.4
	MC1	0.9	4	19.7	69.8	156.1	248.2
	MC5	0.8	4	20.2	73.8	167.4	252.8
	MC10	0.8	3.7	19.3	71.6	166.9	253.1
	MC50	0.8	4	19.7	70.5	163.2	249.8
45	A	1	5	24	79.7	176.8	262.7
	B	2.1	8.1	29.7	78.3	153.6	219.3
	MC1	1.2	5.2	26.5	90.9	186.6	256.1
	MC5	1	4.9	23.9	77.4	173.4	258.6
	MC10	1	5.2	24.3	80.9	174.4	255.5
	MC50	1	5	24.1	80.5	176	260.5
50	A	1.3	6.2	28.4	88.1	186.9	273.6
	B	2.6	10	34.6	85.8	163.4	230.6
	MC1	1.2	7	29.9	86	181.8	268.4
	MC5	1.3	6.1	28.2	86.4	182.1	272.3
	MC10	1.4	6.5	29.4	89.8	192.2	279.4
	MC50	1.3	6.2	28.2	88.2	186.5	273.3
55	A	1.6	7.7	33.2	96.1	196.4	283.5
	B	3.3	12.3	39.7	93.2	172.8	241.3
	MC1	1.5	8.3	34.1	95.7	203.3	282.2
	MC5	1.6	7.9	34.2	95.7	195.6	300.1
	MC10	1.6	7.3	32.2	93.9	197.7	286.6
	MC50	1.6	7.6	33.1	95.5	195.7	282.8
60	A	2.1	9.5	38.3	103.8	205.1	292.6
	B	4.2	14.9	44.9	100.4	181.7	251.5
	MC1	2.5	11.3	40.9	109.9	216.9	286.2
	MC5	2.3	9.8	39.2	104.2	209.3	296
Quantiles							
Age, y	Model	0.1	0.25	0.5	0.75	0.9	0.95
	MC10	2.3	10.2	40.3	110.6	213.8	301.5
	MC50	2.1	9.6	38.1	103.6	205.9	293
65	A	2.6	11.6	43.5	111.1	213.1	300.7
	B	5.2	17.8	50.2	107.2	190.1	261
	MC1	2.3	11	42.4	115	237.4	302.9
	MC5	2.8	12.5	45.4	112.6	208.4	305.2
	MC10	2.7	11.9	44.9	111.4	214.5	299.9
	MC50	2.6	11.6	43.2	110.7	211.5	303

Models A and B estimate the quantiles of weekly alcohol intake distribution based on the numerical method proposed in the paper. Model A adjusts for the correlation between *probability* and *amount* parts; Model B assumes zero correlation between the *probability* and *amount* parts. Models MC1, MC5, MC10 and MC50 estimate the quantiles of weekly alcohol intake distribution under the assumptions of model A based on Monte Carlo simulations. The estimates of MC1 are based on 1000 observations, MC5 on 5000 observations, MC10 on 10000 observations and MC50 on 50000 observations per covariate pattern

#### Comparison with Monte Carlo simulation

Table 3 shows the results of Monte Carlo simulation (model A only), based on 1000, 5000, 10,000 and 50,000 simulated datasets for a given covariate pattern.

Monte Carlo simulation estimates show better convergence to the estimates obtained via the numerical method with increasing number of simulations. The difference between results is more pronounced at the tails of the distribution. For example, for a group of 45-year-old men, the 0.95 quantile obtained from the Monte Carlo simulated dataset of 1000 observations is equal to 391.5 g, which is considerably lower than 414.9 g obtained from the suggested numerical approach and compared to 414.0 g obtained when increasing the number of datasets to 50,000.

#### Adherence to maximum recommended intake

The proposed method also allows the estimation of the percentage of participants who adhere to the current recommendations with respect to reference intakes. For example, the Department of Health [10] recommends that maximum daily alcohol intake should not exceed 32 g for men and 24 g for women, which accumulates to weekly maximum intake of 224 g for men and 168 g for women. Applying the method described in this paper we estimate that among 45-year-old participants 21 % of males and 11 % of females exceed the maximum recommended weekly alcohol intake.

## Discussion

The paper utilises the two-part mixed-effects model introduced by [23] and followed by [30], and extends the work by [32] by suggesting a concise numerical method, as an alternative to Monte Carlo simulations, for the estimation of the distribution of occasionally consumed foods in specified population sub-groups. We show that, although quantile estimates obtained with simulations converge to numerically obtained estimates, the number of simulated observations needed per covariate pattern cannot be known in advance and depends on the structure of the data at hand. With the differences between the estimates obtained from both methods most pronounced at the tails of the distribution, the method can be especially applicable when the focus of research is under- or over-consumption of certain nutrients, foods or beverages. Furthermore, since the method is faster than simulations, it is especially convenient when the number of covariate patterns is large.

There are several extensions to the two-part mixed-effects model, as [23] show, it may include random slopes in addition to the random intercepts used here, thus widening their application to more complex study designs, such as longitudinal studies. Tooze et al. [32] suggested transforming the original recorded amount of food consumed based not only on the log-normal distribution, but also including Box-Cox power transformations. Consequently the back-transformations to the original scale of the continuous response is required [22]. Liu et al. [17] suggested to use the generalised gamma distribution for continuous positive responses. Furthermore, [27] discussed in depth the bias, arising in regression coefficients, when the correlation between the model parts is not accounted for. Our results provide an illustration of the impact of this form of model specification on the estimated distribution of alcohol intake. Further [28,29] suggest the bridge distribution for the random effect in the *probability* part of the model and provide extensive discussion on interpretation of the marginal effects of the two-part model.

Often, it is also of interest to investigate the relationship between predicted dietary intake and health outcomes. We have showed that the between-person variation of alcohol consumption can be substantial. Therefore, when utilising predicted values of intake in relationship with health outcomes, this variation should be taken into account.

There are several limitations of the described model and the proposed method. First, it is assumed that all consumed foods are reported (i.e. the reported intake is an unbiased measure of the true intake on the original or transformed scale), which might be unlikely for some subgroups of people as demonstrated by doubly labelled water studies [31]. We tried to minimise the potential bias by excluding those with high degree of energy under-reporting. However, if misreporting is present then the estimated intake distribution can also be biased.

The two-part model allows the probability to consume to be very small but not zero, so we cannot distinguish never- from rare-consumers. Keogh [15] suggests a model extension to adjust for never-consumers.

We limited the applicability of the model to natural logarithm transformed data to obtain symmetry in the shape of the distribution, which might be too restrictive in some cases. We also did not incorporate weights for if the data are obtained from surveys and the generalisation to the whole population is required. These, along with the extension of the method to the estimation of intake of multiple correlated foods is the area of further research.

## Conclusions

In summary, this paper provides a new numerical method for the concise estimation of occasionally consumed food intake distribution within a specified sub-population. The method is based on estimates obtained from the two-part mixed-effects model and utilises numerical integration and optimisation techniques which can be readily implemented. It is less time consuming than simulation based method, which is especially beneficial for when the number of the predictors of food intake is large. It does not rely on simulation so the precision of quantiles estimates does not depend on simulated data size. We hope that this work will encourage the application of the two-part mixed-effects model in the wider research community as it shows that the model is very flexible and can incorporate various explanatory factors such as seasonality, the day of the week, gender, age, behavioural and socio-economic status. Incorporating relevant explanatory factors reduces the between-person variation and thus can help uncover potential causal relationships between food intake and social, environmental, personal and behavioural predictors. This is a very active area of current nutrition research.

## Abbreviations

FFQ, food frequency questionnaire; FD, food diary; 24HR, 24 hour food recall; RCT, randomised control trial; BMI, body mass index

## Additional files

Additional file 1 **Appendix 1.** Provides theoretical details on the suggested method. (PDF 141 kb)

Additional file 2 **Appendix 2.** Briefly describes the methods employed when estimating under-reporting in the specified population. (DOC 17 kb)

## References

[1] Albert PS (2005). Letter to the editor. Biometrics.

[2] Ashfield-Watt P, Welch AA, Day NE, Bingham SA (2004). Is ‘five-a-day’ an effective way of increasing fruit and vegetable intakes?. Public Health Nutr.

[3] Beaton GH, Milner J, Corey P, McGuire V, Cousins M, Stewart E, de Ramos M, Hewitt D, Grambsch PV, Kassim N, Little JA (1979). Sources of variance in 24-hour dietary recall data: implications for nutrition study design and interpretation. Am J Clin Nutr.

[4] Bingham SA, Gill C, Welch A, Day K, Cassidy A, Khaw KT, Sneyd MJ, Key TJA, Roe L, Day NE (1994). Comparison of dietary assessment methods in nutritional epidemiology: weighed records v. 24 h recalls, food-frequency questionnaires and estimated-diet records. Br J Nutr.

[5] Bingham SA, Gill C, Welch A, Cassidy A, Runswick SA, Oakes S, Lubin R, Thurnham DI, Key TJ, Roe L, Khaw KT, Day NE (1997). Validation of dietary assessment methods in the UK arm of EPIC using weighed records, and 24-hour urinary nitrogen and potassium and serum vitamin C and carotenoids as biomarkers. Int J Epidemiol.

[6] Black AE (2000). Critical evaluation of energy intake using the goldberg cut-off for energy intake:basal metabolic rate. a practical guide to its calculation, use and limitations. Int J Obes.

[7] Braam LAJLM, Ocké MC, Bueno-de-Mesquita HB, Seidell JC (1998). Determinants of obesity-related underreporting of energy intake. Am J Epidemiol.

[8] Burrows TL, Martin RJ, Collins CE (2010). A systematic review of the validity of dietary assessment methods in children when compared with the method of doubly labeled water. J Am Dietetic Assoc.

[9] Day NE, McKeown N, Wong MY, Welch A, Bingham S (2001). Epidemiological assessment of diet: a comparison of a 7-day diary with a food frequency questionnaire using urinary markers of nitrogen, potassium and sodium. Int J Epidemiol.

[10] Department of Health, Ellison J. Policy. Harmful drinking. 2013. https://www.gov.uk/government/policies/reducing-harmful-drinking. Accessed 1 June 2015.

[11] Dodd KW, Guenther PM, Freedman LS, Subar AF, Kipnis V, Midthune D, Tooze JA, Krebs-Smith SM (2006). Statistical methods for estimating usual intake of nutrients and foods: A review of the theory. J Am Diet Assoc.

[12] Freedman LS, Guenther PM, Krebs-Smith SM, Dodd KW, Midthune D (2010). A population’s distribution of healthy eating index-2005 component scores can be estimated when more than one 24-hour recall is available. J Nutrition.

[13] Guenther PM, Dodd KW, Reedy J, Krebs-Smith SM (2006). Most americans eat much less than recommended amounts of fruits and vegetables. J Am Diet Assoc.

[14] Jebb SA, Lovegrove JA, Griffin BA, Frost GS, Moore CS, Chatfield MD, Bluck LJ, Williams CM, Sanders TAB (2010). Effect of changing the amount and type of fat and carbohydrate on insulin sensitivity and cardiovascular risk: the risck (reading, imperial, surrey, cambridge, and kings) trial. Am J Clin Nutr.

[15] Keogh R (2011). Allowing for never and episodic consumers when correcting for error in food record measurements of dietary intake. Biostatistics.

[16] Kipnis V, Midthune D, Buckman DW, Dodd KW, Guenther PM, Krebs-Smith SM, Subar AF, Tooze JA, Carroll RJ, Freedman LS (2009). Modeling data with excess zeros and measurement error: Application to evaluating relationships between episodically consumed foods and health outcomes. Biometrics.

[17] Liu L, Cowen ME, Strawderman RL, Shih Y-CT (2010). A flexible two-part random effects model for correlated medical costs. J Health Econ.

[18] McKeown NM, Day NE, Welch AA, Runswick SA, Luben RN, Mulligan AA, McTaggart A, Bingham SA (2001). Use of biological markers to validate self-reported dietary intake in a random sample of the european prospective investigation into cancer united kingdom norfolk cohort. Am J Clin Nutri.

[19] Mendez MA, Popkin BM, Buckland G, Schroder H, Amiano P, Barricarte A, Huerta JM, Quirós JR, Sánchez MJ, González CA (2011). Alternative methods of accounting for underreporting and overreporting when measuring dietary intake-obesity relations. Am J Epidemiol.

[20] Nelson M, Black AE, Morris JA, Cole TJ (1989). Between- and within-subject variation in nutrient intake from infancy to old age: estimating the number of days required to rank dietary intakes with desired precision. Am J Clin Nutr.

[21] Nusser SM, Fuller WA, Guenther PM (1987). Estimating Usual Dietary Intake Distributions: Adjusting for Measurement Error and Non-normality in 24-hour Food Intake Data. Survey Measurement and Process Quality.

[22] Nusser SM, Carriquiry AL, Dodd KW, Fuller WA (1996). A semiparametric transformation approach to estimating usual daily intake distributions. J Am Stat Assoc.

[23] Olsen MK, Schafer JL (2001). A two-part random-effects model for semicontinuous longitudinal data. J Am Stat Assoc.

[24] Rosenbaum M, Ravussin E, Matthews DE, Gilker C, Ferraro R, Heymsfield SB, Hirsch J, Leibel RL (1996). A comparative study of different means of assessing long-term energy expenditure in humans. Am J Physiol.

[25] Rutishauser I, Black A, Gibney MJ, Vorster HH, Kok FJ (2002). Measuring food intake. Introduction to Human Nutrition.

[26] Sempos CT, Johnson NE, Smith EL, Gilligan C (1985). Effects of intraindividual and interindividual variation in repeated dietary records. Am J Epidemiol.

[27] Su L, Tom BDM, Farewell VT (2009). Bias in 2-part mixed models for longitudinal semicontinuous data. Biostatistics.

[28] Su L, Tom BDM, Farewell VT (2011). A likelihood-based two-part marginal model for longitudinal semi-continuous data. Stat Methods Med Res.

[29] Tom BDM, Su L, Farewell VT. A corrected formulation for marginal inference derived from two-part mixed models for longitudinal semi-continuous data. Stat Methods Med Res. 2013;:1–7. doi:10.1177/0962280213509798. 10.1177/0962280213509798 PMC5051603 24201470

[30] Tooze JA, Grunwald GK, Jones RH (2002). Analysis of repeated measures data with clumping at zero. Stat Methods Med Res.

[31] Tooze JA, Subar AF, Thompson FE, Troiano R, Schatzkin A, Kipnis V (2004). Psychosocial predictors of energy underreporting in a large doubly labeled water study. Am J Clin Nutr.

[32] Tooze JA, Midthune D, Dodd KW, Freedman LS, Krebs-Smith SM, Subar AF, Guenther PM, Carroll RJ, Kipnis V (2006). A new statistical method for estimating the usual intake of episodically consumed foods with application to their distribution. J Am Diet Assoc.

[33] Tooze JA, Kipnis V, Buckman DW, Carroll RJ, Freedman LS, Guenther PM, Krebs-Smith SM, Subar AF, Dodd KW (2010). A mixed-effects model approach for estimating the distribution of usual intake of nutrients: The NCI method. Stat Med.

[34] Wald NJ (2007). Folic acid and neural tube defects: The current evidence and implications for prevention. Neural Tube Defects.

[35] Xiao X, White EP, Hooten MB, Durham SL (2011). On the use of log-transformation vs. nonlinear regression for analyzing biological power laws. Ecology.

[36] National Institute for Health Research. School for Public Health Research. School-wide programmes. Alcohol. http://sphr.nihr.ac.uk/research/school-wide-programmes/. Accessed 28 April 2015.

