# Supplementary material for: A simplified approach to estimating the distribution of occasionally-consumed dietary components, applied to alcohol intake
Source: BMC Med Res Methodol. 2016 Jul 1;16:78. doi: 10.1186/s12874-016-0178-3 (PMC4930587; doi:10.1186/s12874-016-0178-3)
Supplement: Supplementary file 1 — Appendix 1. Provides theoretical details on the suggested method. (PDF 141 kb) [file 12874_2016_178_MOESM1_ESM.pdf]

Appendix 1

We show that the solution to equation (3) in the Methods section, defining the quantile c_p, is unique. This involves the integral

$$I(c) = \int_{-\infty}^{+\infty} \Big(\int_{-\infty}^{h(c,v_i)} f_{BN}(u_i, v_i) du_i \Big) dv_i,$$

where $f_{BN}(u_i, v_i)$ denotes the probability density function of the bivariate normal distribution with mean 0 and covariance matrix

$$\Sigma = \begin{pmatrix} \sigma_u^2 & \rho\sigma_u\sigma_v \\ \rho\sigma_u\sigma_v & \sigma_v^2 \end{pmatrix},$$

ρ is the correlation between u_i and v_i and σ_u, σ_v are the corresponding standard deviations of u and v. Consider the function $h(c, v_i)$

$$h(c, v_i) = \ln(c) - \ln \Bigg(4 \exp(x_{i0}^{'}\tilde{\beta} + u_i + 0.5\sigma_\epsilon^2) \frac{\exp(x_{i0}^{'}\tilde{\gamma} + v_i)}{1 + \exp(x_{i0}^{'}\tilde{\gamma} + v_i)} + 3 \exp(x_{i1}^{'}\tilde{\beta} + u_i + 0.5\sigma_\epsilon^2) \frac{\exp(x_{i1}^{'}\tilde{\gamma} + v_i)}{1 + \exp(x_{i1}^{'}\tilde{\gamma} + v_i)} \Bigg).$$

Then under the above assumptions the following holds true:

$h(c, v_i)$ is continuous for all $c > 0$ and for all v_i,

$h(c, v_i)$ is strictly decreasing in v_i, $\frac{\partial h(c,v_i)}{\partial v_i} < 0$ for all v_i,

$h(c, v_i)$ is strictly increasing in c, $\frac{\partial h(c,v_i)}{\partial c} > 0$ for all c,

As $f_{BN}(u_i, v_i)$ is strictly positive and continuous by definition, it follows that $I(c)$ is continuous and strictly increasing with c. Moreover, as $f_{BN}(u_i, v_i)$ is a probability density function, the following holds: $0 < I(c) < 1$ for all $c > 0$ and both the upper and the lower bounds for $I(c)$ are tight in the sense that $\lim_{c \to 0} I(c) = 0$, and $\lim_{c \to \infty} I(c) = 1$. Hence, by applying the intermediate value theorem [1] to $I(c)$, it follows that for all $p \in (0, 1)$ there exists a *unique* quantile at level p, c_p, that solves equation $I(c_p) = p$. The uniqueness follows from the strict monotonicity of $I(c)$.

Reference

1. Garling, D.J.H.: A Course in Mathematical Analysis vol. 1, CUP, Cambridge, 2013.
