# Supplementary material for: A simplified approach to estimating the distribution of occasionally-consumed dietary components, applied to alcohol intake
Source: BMC Med Res Methodol. 2016 Jul 1;16:78. doi: 10.1186/s12874-016-0178-3 (PMC4930587; doi:10.1186/s12874-016-0178-3)
Supplement: Supplementary file 2 — Appendix 2. Briefly describes the methods employed when estimating under-reporting in the specified population. (DOC 17 kb) [file 12874_2016_178_MOESM2_ESM.doc]

Appendix 2

***Under-reporters***

The analysis required careful consideration of under-reporting. A brief summary of the information used to identify extreme under-reporters in this study is presented. The status of “under-reporter” was defined by the Goldberg cut-off (1) and based on the following information: the within-person coefficient of variation of the total Kcal intake of 24%, the number of dietary records of, on average, 4 days per person, Basal Metabolic Rate (BMR) estimated by Schofield equations(2), Physical Activity Level (PAL) obtained from the WHO recommendations for energy requirements and assigned to be 1.53 to represent sedentary or lightly active lifestyle (3). Reported energy intake (rEI) was calculated as individual average of reported total energy intake over available record days and estimated energy requirement was estimated as a product of estimated BMR and PAL. To exclude extreme cases of under-reporting, status of "under-reporter" was assigned to everyone whose degree of under-reporting (rEI:EER) was below 0.75 and "adequate" reporters to those whose degree of under-reporting was greater than or equal to 0.75.

**References**

1. Black AE: **Critical evaluation of energy intake using the Goldberg cut-off for energy intake:basal metabolic rate. A practical guide to its calculation, use and limitations**. *International Journal of Obesity* 2000, **24**:1119-1130
2. Schofield W, Schofield C, James WPT: **Basal metabolic rate – review and prediction, together with an annotated bibliography of source material.** *Hum Nutr Clin Nutr* 1985; **39C Suppl 1**
3. **Report of a Joint FAO/WHO/UNU Expert Consultation. Human energy requirements**. In: *Food and Nutrition Technical Report Series.* Rome; 2001.
